# *In Silico* Risk Assessment of HLA-A*02:06-Associated Stevens-Johnson Syndrome and Toxic Epidermal Necrolysis Caused by Cold Medicine Ingredients

**DOI:** 10.1155/2013/514068

**Published:** 2013-10-12

**Authors:** Hideto Isogai, Hiroko Miyadera, Mayumi Ueta, Chie Sotozono, Shigeru Kinoshita, Katsushi Tokunaga, Noriaki Hirayama

**Affiliations:** ^1^Basic Medical Science and Molecular Medicine, Tokai University School of Medicine, 147 Shimokasuya, Isehara, Kanagawa 259-1143, Japan; ^2^Department of Human Genetics, School of International Health, Graduate School of Medicine, The University of Tokyo, 7-3-1 Hongo, Bunkyo-ku, Tokyo 113-0033, Japan; ^3^Department of Ophthalmology, Kyoto Prefectural University of Medicine, Hirokoji Kawaramachi, Kamigyo-ku, Kyoto 602-0841, Japan

## Abstract

Stevens-Johnson syndrome (SJS) and toxic epidermal necrolysis (TEN) are severe drug hypersensitivities with high mortality. Typical over-the-counter drugs of cold medicines are suggested to be causative. As multiple ingredients are generally contained in cold medicines, it is of particular interest to investigate which ingredients are responsible for SJS/TEN. However, experimental examination of causal relationships between SJS/TEN and a particular drug molecule is not straightforward. Significant association between HLA-A*02:06 and SJS/TEN with severe ocular surface complications has been observed in the Japanese. In the present study, we have undertaken *in silico* docking simulations between various ingredients contained in cold medicines available in Japan and the HLA-A*02:06 molecule. We use the composite risk index (CRI) that is the absolute value of the binding affinity multiplied by the daily dose to assess the potential risk of the adverse reactions. The drugs which have been recognized as causative drugs of SJS/TEN in Japan have revealed relatively high CRI, and the association between SJS/TEN and HLA-A*02:06 has been qualitatively verified. The results have also shown that some drugs whose links to SJS/TEN have not been clinically recognized in Japan show the high CRI and suggested that attention should be paid to their adverse drug reactions.

## 1. Introduction

Adverse drug reactions (ADRs) are a serious public health problem. Drug hypersensitivity which constitutes a major category of ADRs is typically severe in nature. In particular, Stevens-Johnson syndrome (SJS) and toxic epidermal necrolysis (TEN) are severe drug hypersensitivities with high mortality. Various drugs have been pointed out as causes of SJS/TEN. In addition to certain prescription drugs, typical over-the-counter drugs of cold medicines are suggested to be causative [1, 2]. These reactions often result in severe and definitive sequelae such as vision loss in some cases [2]. The reported incidence of ocular complications in SJS/TEN is 50–68% [3].

Considering the fact that cold medicines are the most widely used over-the-counter drugs, it is extremely important to know the potential causative ingredients of SJS/TEN. Although nonsteroidal anti-inflammatory drugs which are major ingredients of cold medicines were suggested as causative drugs [2], no systematic studies about drug hypersensitivities of other ingredients have been undertaken. Since it is possible that certain ingredients might cause more severe adverse reactions, predicting the potential adverse reactions of other ingredients will be undoubtedly beneficial to avoid the latent ADRs.

Significant associations between certain ADRs and specific alleles of human leukocyte antigen (HLA) have been pointed out [4]. Recently, the detailed molecular mechanism underlying the strong association between HLA-B*57 and the hypersensitivity of reverse-transcriptase inhibitor abacavir has been disclosed [5]. The relevant adverse drug reaction is triggered by the strong binding of abacavir into the antigenic peptide binding groove of the HLA molecule. It has been reported that the frequency of carriers of the HLA-A*02:06 antigen is significantly higher among Japanese patients with severe ocular surface complications than in other populations [6]. It is highly possible that the adverse drug reactions associated with HLA-A*02:06 are also triggered by the direct interactions between the causative drug molecules and the HLA molecule.

However, it is generally intractable to ascertain experimentally the causation between a specific drug and an incidence of a particular adverse drug reaction especially in cases where multiple drugs are administered simultaneously such as cold medicines. *In silico* analysis based on the underlying molecular mechanism of the ADRs can be employed as a powerful alternative method in these situations. In this study, *in silico* analysis using docking simulations between various ingredient molecules in cold medicines and the HLA-A*02:06 molecule has been undertaken in order to predict what ingredient molecules might cause SJS/TEN through the interactions with HLA-A*02:06.

## 2. Methods

We examined all ingredients contained in four popular over-the-counter cold medicines available in Japan, that is, Lulu, Pabron, Contac, and Benza Block. The ingredient molecules used in docking simulations are given in Table 1. As Chinese herbs contained in all of these over-the-counter cold medicines are mixtures of various agents whose molecular structures are not fully identified, they are excluded from the docking simulations. Lysozyme and vitamins are also excluded. A software system molecular operating environment (MOE) [7] was used throughout this study and all the calculations were performed on a DELL PC workstation T7500.

**Figure 1:**
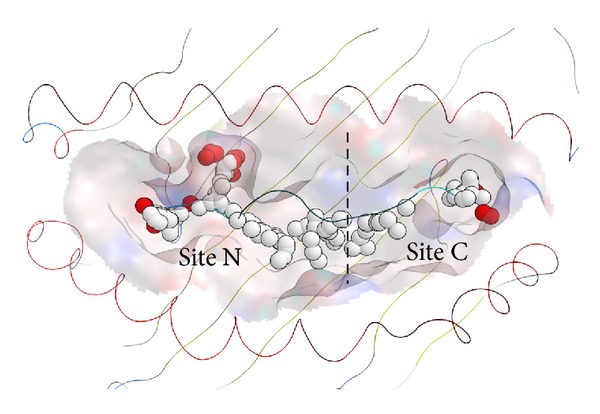
The antigenic-peptide binding groove of HLA-A*02:06 is depicted schematically with the two alpha helices represented by helical lines running horizontally. The white and red alpha spheres calculated in the groove represent hydrophobic and hydrophilic positions, respectively. The binding groove is divided into sites C and N.

The X-ray structure of the HLA-A*02:06 molecule deposited in the Protein Data Bank [8] (PDB ID: 3OXR) was used for docking simulations. It is highly possible that the molecules shown in Table 1 bind to the antigenic-peptide binding groove located between two *α* helices as in the case of abacavir. Since the groove is relatively wide, we have specified the possible binding sites by use of the alpha site finder function [9] implemented in MOE. Small spheres named alpha spheres shown in Figure 1 correspond to locations of tight atomic packing at the antigenic-peptide binding groove. A site where the alpha spheres are clustered is designated alpha site which is considered to be the potential binding site of drug molecules. Two alpha sites designated sites N and C were identified in the antigenic-peptide binding groove of the HLA-A*02:06 molecule and these two sites were considered in docking simulations. All docking simulations were undertaken by use of software ASEDock [10]. ASEDock based on unique concept of ASE model and ASE score uses the alpha sites for docking the small molecule. Since ASEDock is free from any bias except for shape, it is one of the very robust docking methods. The binding affinity of a drug molecule to the HLA molecule was judged by scoring functions of GBVI/WSA_dG [11] which is considered to express protein-ligand binding free energy.

**Figure 2:**
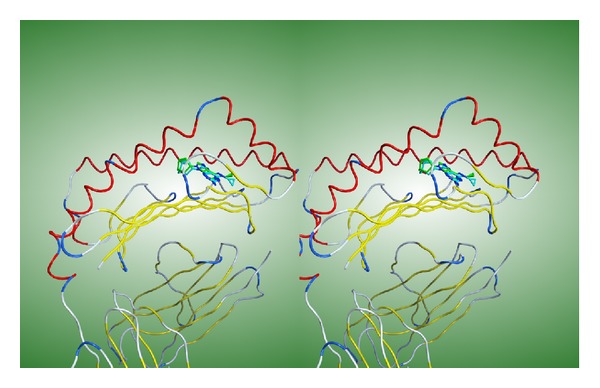
A superposition of simulated and experimental structures of abacavir. The drug is bound at the antigenic-peptide binding groove of the HLA-B*57:01 molecule. The HLA molecules are depicted schematically. The alpha helix and beta strand are shown by red and yellow tubes, respectively. The drug molecules are depicted by stick models. The carbon atoms of abacavir located by X-ray analysis and docking simulations are colored in cyan and green, respectively. This figure is a cross-eyed stereoscopic drawing.

Redocking simulations were performed using the crystal structure of the complex between abacavir and the HLA-B*57:01 molecule (PDB ID: 3VRI) to assess the appropriateness of using ASEDock for the drug-HLA systems. The root-mean square deviation (rmsd) between nonhydrogen atoms of abacavir in the crystal and docked structures is 0.99 Å. Prediction within rmsd of 2.0 Å is held as the passing standard. The validation clearly indicated that ASEDock can simulate accurately enough the structure and position of the bound abacavir molecule in the crystal structure and the docking algorithm is suitable for the docking simulations of the drug-HLA systems. A superposition of the simulated and experimental structures is shown in Figure 2. The GBVI/WSA dG value is calculated to be −6.56 kcal/mol.

## 3. Results and Discussion

The binding affinities to both sites are given in Table 2. As the racemates of methylephedrine and chlorpheniramine are used, the optical isomers with higher binding affinities are given in the table. Although the binding affinity to the HLA molecule would play an important role to trigger the following immunological response, the probability of occurrence of the adverse reactions might significantly depend on the dose of the drug. Therefore it may be reasonable to use a composite risk index (CRI) calculated by multiplying the absolute value of the binding affinity by the daily dose together to assess the potential risk of the adverse reactions. In Table 2, the CRI is given and the drug molecules are sorted by descending order of the CRI calculated from the binding affinity to the site C. The maximum dose of each drug per day in mmol was calculated according to the dose written in each package insert of cold medicines. For comparison, the binding affinity of abacavir to the HLA-B*57 molecule is given. Most of the drugs are preferentially bound at the site N. Drugs with higher CRIs are, however, inclined to bind preferentially at the site C as abacavir.

If we set a CRI threshold of, for example, 1.0 mmol·kcal/mol arbitrarily, nine drugs are judged to be high-risk drugs causing adverse drug reactions through binding to HLA-A*02:06. In Japan, warnings about potential side effects of SJS and TEN are explicitly stated in the ethical drug package inserts of acetaminophen, ibuprofen, and loxoprofen [12]. Cases of SJS/TEN caused by acetaminophen [13], ibuprofen [14], and loxoprofen [15] have been reported, albeit the associations with HLA-A*02:06 have not been noticed. Ethenzamide was reported as a causative drug of SJS in Japan [16]. Since it is highly possible that the SJS caused by these four drugs is associated with HLA-A*02:06, studies to verify the associations are urgently required to understand the molecular mechanisms of the adverse reactions and find countermeasures against them. As to tranexamic acid, guaifenesin, guaiacol, pseudoephedrine, and caffeine, they are not considered as significant causative drugs of SJS at least in Japan. However,17, 4, and 2 SJS cases caused by guaifenesin, pseudoephedrine, and caffeine, respectively, have been reported from the FDA [17]. TEN caused by tranexamic acid was reported [18]. Although it is highly possible that these adverse drug reactions are associated with different alleles, rather high CRIs of these drugs imply the involvement of these drugs in SJS/TEN. As comprehensive analysis about causative factors of SJS has not been undertaken, unknown factors might be involved in the SJS cases. Under these circumstance, we believe that SJS-like symptoms caused by drugs with the high CRIs should be monitored carefully.

The drugs with the CRI being lesser than 1.0 have not been noticed as culprit drugs for SJS/TEN at least in Japan. The low incidence of SJS/TEN caused by these drugs in Japan supports that the SJS/TEN associated with HLA-A*02:06 should be mainly determined by CRI. Although certain numbers of SJS cases have been reported on drugs such as clemastine, mequitazine and bromhexine from the FDA, the causative alleles in these cases may be different from HLA-A*02:06. Hypersensitivities against these drugs are described in the ethical drug package inserts in Japan.

Metabolites for each drug molecule have not been fully disclosed yet. It is possible that some of such metabolites rather than the parent drugs would play more important roles in certain cases of ADRs. Therefore further simulation studies including the metabolites will be essential and interesting in the more distant future. Nevertheless, these uncertainties do not preclude the usefulness of the simulation results obtained for the parent drugs in the context of a weight of evidence approach for the time being. The *in silico* risk assessment methodology as used in the present study will certainly play an important role in identifying testing needs, setting testing priorities, and above all attracting our attention to the potential toxicities of the relevant drugs.

**Figure 3:**
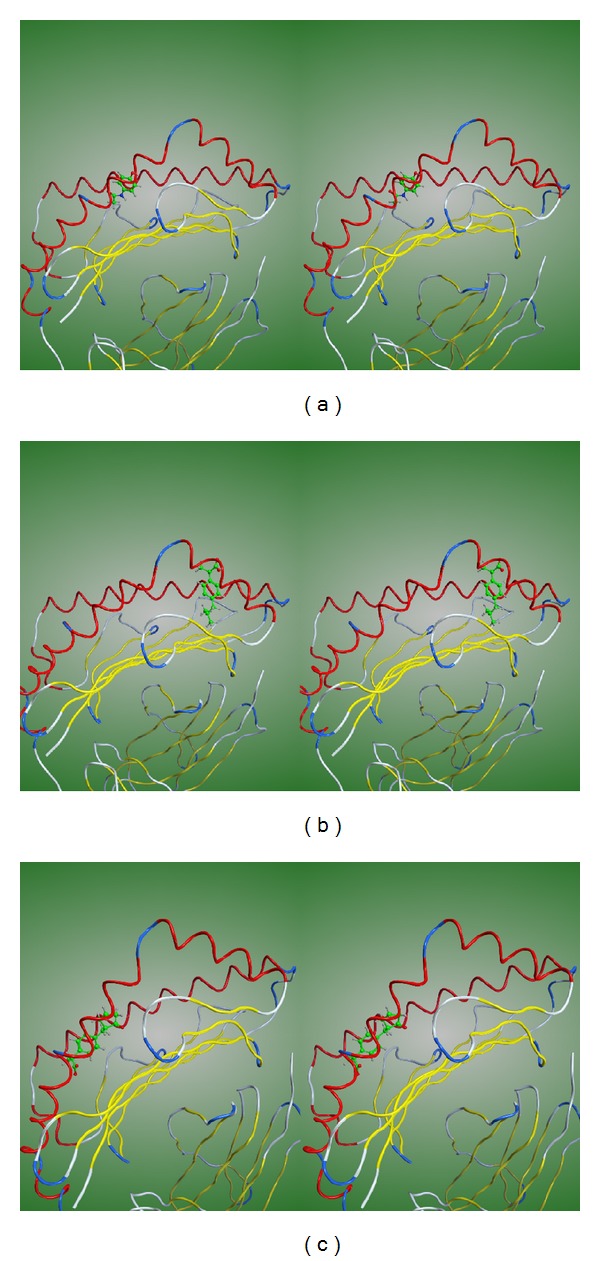
Cross-eyed stereoscopic drawings of the drugs bound at the antigenic-peptide binding grooves of ADR-associated HLA molecules. The drugs are represented by stick models with carbon atoms and bonds colored in green. The interaction modes of acetaminophen, ibuprofen, and loxoprofen at the peptide-binding groove of the HLA-A*02:06 molecule are shown in (a), (b), and (c), respectively.

The binding modes of acetaminophen, ibuprofen, and loxoprofen at the antigenic peptide-binding groove of the HLA-A*02:06 molecule are illustrated in Figures 3(a), 3(b), and 3(c), respectively. The binding mode of abacavir to the HLA-B*57:01 is shown in Figure 2. In the crystal structure [5], abacavir binds deep across the bottom of the antigen-binding groove of the HLA molecule. Therefore new endogenous peptides can bind on top of the bound abacavir leading to the adverse drug reaction of abacavir. On the other hand, acetaminophen and loxoprofen are bound near the surface of the HLA molecule. Although one end of the ibuprofen molecule is bound to the bottom of the groove, the carboxyl group is exposed on the surface of the HLA molecule. Since the binding modes of these drugs appear different from that of abacavir, it is not clear whether new endogenous peptides can bind on top of the bound drugs. Hence further studies to confirm the molecular mechanisms leading to the ADRs are required.

## 4. Conclusions

Prompt recovery from SJS/TEN upon withdrawal of the relevant drugs indicates the importance of early diagnosis. However, identification of the culprit drugs is usually intractable especially in the cases where patients take drugs with multiple ingredients such as cold medicines. In these situations, a comprehensive risk assessment taking both of circumstantial evidence and theoretically deduced risk index into account would be helpful. In the present study we have shown that docking simulations between drug molecules and the HLA-A*02:06 molecule can explain the episodes of culprit drugs of SJS/TEN reported so far in Japan. In addition, the present study has pointed out the potential risk of several drugs whose involvement in SJS/TEN has not been explicitly noticed until now. This study urges further investigations of verifying the ADRs of these drugs in detail.

## Figures and Tables

**Table 1:** Ingredients contained in popular cold medicines available in Japan. Chinese herbs, vitamins, and lysozyme are excluded from the table.

Drug name	Therapeutic category
Acetaminophen	Analgesic; antipyretic
Ambroxol	Expectorant
Bromhexine	Expectorant; mucolytic
Caffeine	CNS stimulant; respiratory stimulant
Carbinoxamine	Antihistaminic
*d*-Chlorpheniramine	Antihistaminic
Clemastine	Antihistaminic
Dextromethorphan	Antitussive
Dihydrocodeine	Analgesic (narcotic); antitussive
Ethenzamide	Analgesic
Guaiacol	Expectorant
Guaifenesin	Expectorant
Ibuprofen	Anti-inflammatory; analgesic; antipyretic
Isopropamide	Antispasmodic
Loxoprofen (*R*, *S*)	Anti-inflammatory; analgesic
Mequitazine	Antihistaminic
*d*-Methylephedrine	Analeptic
Noscapine	Antitussive
Pseudoephedrine	Decongestant
Tranexamic acid	Hemostatic

**Table 2:** Binding affinities of the ingredients of cold medicines to the antigenic-peptide binding groove of HLA-A*02:06. Composite risk index is calculated by multiplying the maximum daily dose and the absolute value of GBVI/WSA_dG.

Drug name	Maximum dose/day (mmol)	GBVI_dG* (site N) (kcal/mol)	GBVI_dG* (site C) (kcal/mol)	Composite risk index (site N)**	Composite risk index (site C)**	Preferential binding site
Ethenzamide	6.356	−4.966	−5.335	31.565	33.914	C
Acetaminophen	5.954	−4.623	−4.608	27.523	27.438	N
Tranexamic acid	4.771	−5.550	−5.613	26.478	26.776	C
Ibuprofen	2.181	−5.491	−5.720	11.979	12.477	C
Abacavir***	1.789		−6.562		11.740	C
Guaifenesin	1.211	−6.482	−5.820	7.848	7.047	N
Guaiacolsulfonate	0.991	−4.562	−5.482	4.519	5.430	C
Pseudoephedrine	0.669	−5.581	−5.348	3.736	3.579	N
Caffeine	0.463	−5.333	−5.128	2.471	2.377	N
Loxoprofen	0.168	−6.468	−6.184	1.086	1.038	N
Dextromethorphan	0.130	−6.537	−6.315	0.847	0.819	N
Noscapine	0.116	−7.464	−6.792	0.867	0.789	N
*d*-Methylephedrine	0.139	−6.029	−5.636	0.838	0.784	N
Ambroxol	0.109	−6.763	−6.247	0.734	0.678	N
Dihydrocodeine	0.060	−5.401	−5.709	0.325	0.343	C
Bromhexine	0.029	−6.602	−5.655	0.192	0.164	N
Carbinoxamine	0.018	−7.616	−6.073	0.140	0.112	N
Isopropamide	0.012	−7.729	−6.102	0.097	0.076	N
*d*-Chlorpheniramine	0.010	−7.265	−6.690	0.070	0.064	N
Mequitazine	0.012	−7.021	−5.021	0.087	0.062	N
Clemastine	0.003	−7.680	−6.265	0.022	0.018	N

*GBVI_dG: GBVI/WSA_dG. **Composite risk index: |GBVI/WSA_dG|  × (maximum daily dose).

***Binding to the HLA-B*57:01 molecule.

## References

[1] Yamane Y, Aihara M, Ikezawa Z (2007). Analysis of Stevens-Johnson syndrome and toxic epidermal necrolysis in Japan from 2000 to 2006. *Allergology International*.

[2] Ueta M, Sotozono C, Nakano M (2010). Association between prostaglandin E receptor 3 polymorphisms and Stevens-Johnson syndrome identified by means of a genome-wide association study. *Journal of Allergy and Clinical Immunology*.

[3] Power WJ, Ghoraishi M, Merayo-Lloves J, Neves RA, Foster CS (1995). Analysis of the acute ophthalmic manifestations of the erythema multiforme/Stevens-Johnson syndrome/toxic epidermal necrolysis disease spectrum. *Ophthalmology*.

[4] Illing PT, Vivian JP, Purcell AW, Rossjohn J, McCluskey J (2013). Human leukocyte antigen-associated drug hypersensitivity. *Current Opinion in Immunology*.

[5] Illing PT, Vivian JP, Dudek NL (2012). Immune self-reactivity triggered by drug-modified HLA-peptide repertoire. *Nature*.

[6] Ueta M, Tokunaga K, Sotozono C (2008). HLA class I and II gene polymorphisms in Stevens-Johnson syndrome with ocular complications in Japanese. *Molecular Vision*.

[7] MOE (Molecular Operating Environment)

[8] Bernstein FC, Koetzle TF, Williams GJB (1977). The protein data bank: a computer based archival file for macromolecular structures. *Journal of Molecular Biology*.

[9] Labute P, Santavy M (2007). Locating binding sites in protein structures. *Journal of Chemical Computing Group*.

[10] Goto J, Kataoka R, Muta H, Hirayama N (2008). ASEDock-docking based on alpha spheres and excluded volumes. *Journal of Chemical Information and Modeling*.

[11] Corbeil CR, Williams CI, Labute P (2012). Variability in docking success rates due to dataset preparation. *Journal of Computer-Aided Molecular Design*.

[12] http://www.info.pmda.go.jp/psearch/html/menu_tenpu_base.html.

[13] Khawaja A, Shahab A, Hussain SA (2012). Acetaminophen induced steven Johnson Syndrome-Toxic epidermal necrolysis overlap. *Journal of the Pakistan Medical Association*.

[14] (2005). Ibuprofen: Stevens-Johnson syndrome. *WHO Drugs Information*.

[15] Nagata H, Harada T, Nariai Y, Matsushima H, Yoshimura Y (2003). Allergic reaction originated from loxofrofen sodium; Report a case. *Journal of The Japanese Stomatological Society*.

[16] Inoue A, Takebayashi R, Shoji A (1999). A case of Stevens-Johnson syndrome due to ethenzamide. *Japanese Journal of Dermatoallergology*.

[17] http://www.fda.gov/Drugs/GuidanceComplianceRegulatoryInformation/Surveillance/AdverseDrugEffects/default.htm.

[18] Irazabal MP, Martin LM, Gil LA, Gastearena MAI (2013). Tranexamic acid-induced toxic epidermal necrolysis. *The Annals of Pharmacotherapy*.

